# Women as Medical Students

**Published:** 1869-12

**Authors:** 


### *Women as Medical Students.*

OUR table is flooded with circulars and rejoinders, remonstrances, petitions, solemn communications and squibs on the woman question. As we propose to publish an exclusively scientific medical journal, we can not afford the space. The woman question, the abnormalities of the code, squabbles about fees, medical colleges, new systems, jeremiads and jubilates, etc., etc., must find some other mouthpiece.

Ladies and gentlemen, excuse us; we mean that this journal shall be *for all*, but not an *omnibus* to lug the above-mentioned passengers into general favor or notoriety.

THANKS to contemporaries who have so kindly noticed the improved issues of the JOURNAL.

